# Construction of a 0D/1D composite based on Au nanoparticles/CuBi_2_O_4_ microrods for efficient visible-light-driven photocatalytic activity

**DOI:** 10.3762/bjnano.10.134

**Published:** 2019-07-04

**Authors:** Weilong Shi, Mingyang Li, Hongji Ren, Feng Guo, Xiliu Huang, Yu Shi, Yubin Tang

**Affiliations:** 1 School of Material Science and Engineering, Jiangsu University of Science and Technology, Zhenjiang, Jiangsu 212003, PR China; 2 School of Environmental and Chemical Engineering, Jiangsu University of Science and Technology, Zhenjiang, Jiangsu 212018, PR China; 3 School of Energy and Power, Jiangsu University of Science and Technology, Zhenjiang, Jiangsu 212003, PR China

**Keywords:** Au nanoparticles, 0D/1D composite, CuBi_2_O_4_ microrods, photocatalysis, photocatalytic degradation

## Abstract

Photocatalysis is considered to be a promising technique for the degradation of organic pollutants. Herein, a 0D/1D composite photocatalyst consisting of Au nanoparticles (NPs) and CuBi_2_O_4_ microrods (Au/CBO) was designed and prepared by a simple thermal reduction–precipitation approach. It shows excellent photocatalytic performance in the degradation of tetracycline (TC). The maximum photocatalytic degradation rate constant for Au/CBO composites with 2.5 wt % Au NPs was 4.76 times as high as that of bare CBO microrods. Additionally, the 0D/1D Au/CBO composite also exhibited ideal stability. The significant improvement of the photocatalytic performance could be attributed to the improved light harvesting and increased specific surface area, enhancing photoresponse and providing more active sites. Our work shows a possible design of efficient photocatalysts for environmental remediation.

## Introduction

Heterogeneous semiconductor photocatalysis as an advanced green technology has been widely studied and applied for the removal of organic pollutants from water [1–3]. The catalytic activity of many wide-bandgap (*E*_g_) semiconductor photocatalysts is restricted to UV light radiation, which is only 5% of the solar spectrum. Hence, the development of visible-light-driven photocatalysts is highly desirable because visible light accounts for about 43% of the solar spectrum.

Currently, bismuth-based semiconductor materials, including Bi_2_O_3_ [4], BiVO_4_ [5–6], Bi_2_WO_6_ [7], Bi_2_MoO_6_ [8], BiOX (X = Cl, Br, I) [9], and Bi_2_O_2_CO_3_ [10], are explored as novel visible-light-driven photocatalysts. Due to the valence-band hybridization of O 2p and Bi 6s, the bismuth-based photocatalysts possess a relatively narrow bandgap. Moreover, benefiting from the filled Bi 6s band of Bi^3+^ or the empty 6s band of Bi^5+^, Bi-containing compounds strongly absorb visible light. Among these compounds, the spinel-type compound CuBi_2_O_4_ has attracted much attention because of its low phonon energy, strong visible-light response and adequate thermal stability [11–12]. Because of these attractive features, CuBi_2_O_4_ has been widely studied as a photocatalytic material in the degradation of organic contaminants in water [13–14]. However, bare CuBi_2_O_4_ shows poor photocatalytic performance under visible-light irradiation because of the rapid recombination rate of photo-induced charge carriers and the low chemical affinity to substrate ions. This results in only a small portion of the carriers migrating to the surface of the semiconductor to participate in the photoreactions [15]. Decorating semiconductors with noble metals, such as Ag, Au, and Pt, is a strategy to enhance the photocatalytic performance. Certain noble metals exhibiting surface plasmon resonance (SPR) can promote the absorption of visible light and produce hot charge carriers that can increase the charge density of the substrate semiconductor [16–17]. In addition, as an effective electron sink, noble metals can capture photogenerated electrons and leave holes on the surface of semiconductors, which can be used for the oxidative degradation of organic pollutants [18]. In this context, there is a need for more efforts regarding the design and development of efficient noble metal-decorated CuBi_2_O_4_ photocatalysts.

Recently, composite photocatalysts of zero-dimensional (0D) and one-dimensional (1D) materials have gained attention due to their interesting geometrical and particular chemical properties [19–22]. These excellent properties are: (i) 1D materials have a long axis for efficient absorption of incident sunlight and short path lengths for the transport of photo-induced charge carriers; (ii) 0D nanomaterials with quantum confinement effect are ideal materials in the field of photocatalysis; (iii) 0D nanomaterials could provide more active sites, further enhancing the separation of photogenerated carriers of the 1D substrate materials; (iv) the intimate bonding of two kinds of dimensional materials promotes the dispersion and stability of 0D nanomaterials. Among the noble metal NPs, Au is considered to be one of the most promising materials because of its high photocatalytic activity, low toxicity and good biocompatibility [23–25]. In addition, size, shape and environment of the Au NPs affect the SPR of Au NPs. The Au NPs promote the rapid separation of charge carriers of semiconductor [10,26–27]. Thus, we attempt to use 0D Au NPs to decorate 1D CuBi_2_O_4_ (CBO) microrods to obtain a new type of efficient 0D/1D composite photocatalyst.

Herein, 0D/1D Au NP/CBO microrod composites were rationally designed and prepared by a facile in situ thermal reduction–precipitation method. The fabricated Au/CBO composites showed a higher photocatalytic activity in the removal of a typical antibiotic (tetracycline, TC, 10 mg/L) under visible-light irradiation (λ > 420 nm) than pristine CBO. Furthermore, a series of characterizations were conducted to explore the enhanced photocatalytic activity of 0D/1D Au/CBO composites in detail.

## Experimental

Rod-like CuBi_2_O_4_ (CBO) was synthesized through a hydrothermal route. Typically, Bi(NO_3_)_3_·5H_2_O (1.358 g), Cu(NO_3_)_3_·3H_2_O (0.668 g) and NaOH (1.68 g) were dissolved in distilled water (70 mL) and constantly stirred for 3 h. Then, the mixed precursor was transferred into a 100 mL steel autoclave and heated for 24 h at 180 °C. The precipitate was washed with distilled water, and dried at 60 °C for 12 h to obtain the CBO microrods. Au nanoparticles were loaded onto the surface of CBO by a facile simple thermal reduction–precipitation method. A certain amount of prepared CBO microrods was added to an aqueous solution of HAuCl_4_·4H_2_O. Afterward, the mixed solution was heated to boiling under continuous stirring for 10 min. The as-prepared product was washed and separated by centrifuged with distilled water, dried at 60 °C for 12 h to obtain the final Au/CBO composite photocatalyst. The mass ratios between Au nanoparticles and CBO microrods were 1%, 1.5%, 2.5%, 3.5% and 5%, named as 1 wt % Au/CBO, 1.5 wt % Au/CBO, 2.5 wt % Au/CBO, 3.5 wt % Au/CBO and 5 wt % Au/CBO, respectively. The photocatalytic experiments and characterizations are given in Supporting Information File 1.

## Results and Discussion

**Figure 1:**
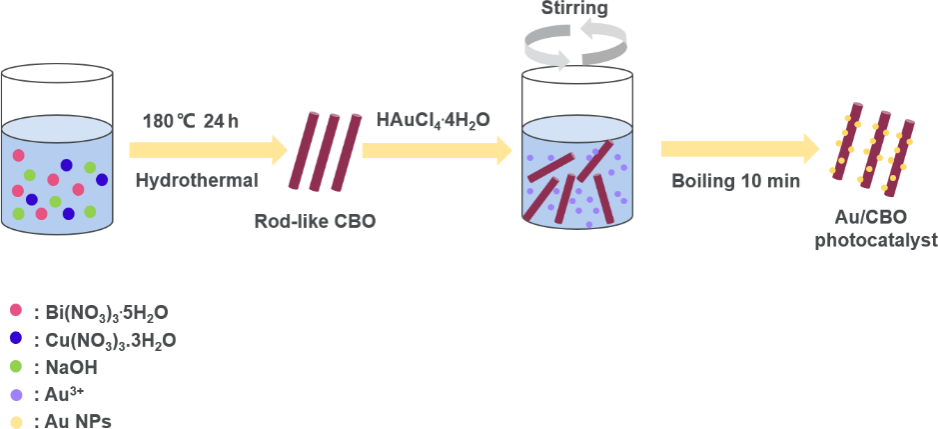
Synthesis of Au/CBO composite.

In this work, we prepared 0D/1D Au/CBO heterostructures via a simple thermal reduction–precipitation method (Figure 1). The rod-like CBO was synthesized through a hydrothermal method and dispersed in aqueous solution of HAuCl_4_. After vigorous stirring, Au^3+^ was adsorbed on the surface CBO microrods. Then, the precursor mixture was boiled to reduce Au^3+^ to Au NPs precipitating on the CBO microrods yielding the Au/CBO hybrid photocatalyst.

**Figure 2:**
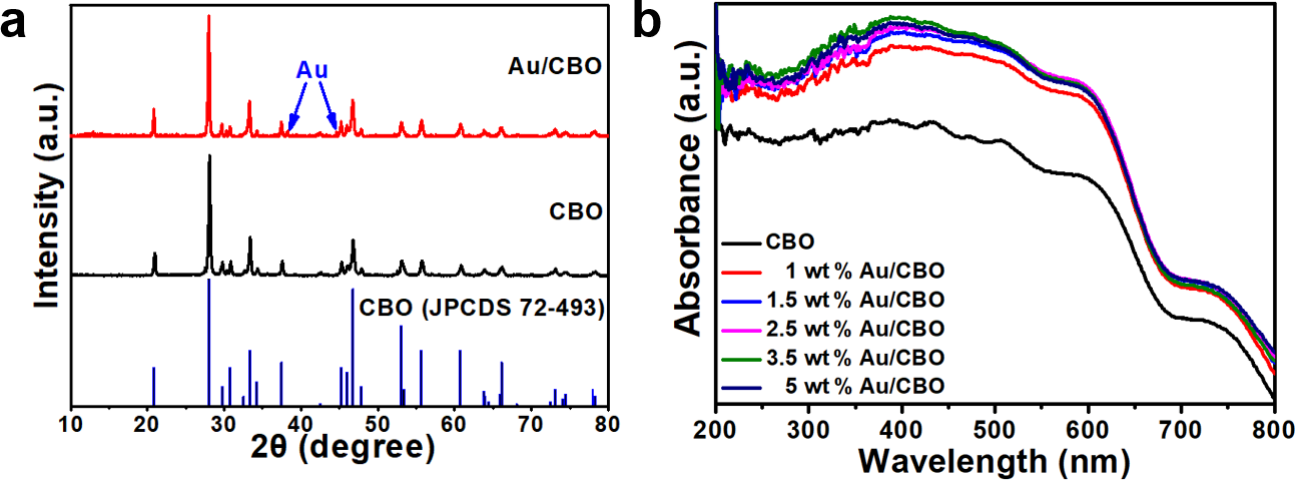
(a) XRD patterns of CBO and 2.5 wt % Au/CBO; (b) UV–vis diffuse reflectance spectra of as-prepared composites.

The crystalline phases of CBO and Au/CBO composite were investigated by XRD (Figure 2a). For pure CBO, all characteristic diffraction peaks can be attributed to the monoclinic phase of CBO (JPCDS 72-493) [28]. After the decoration with Au NPs (2.5 wt % Au/CBO), there are two additional diffraction peaks at 38.2° and 44.4° that can be indexed to the (111) and (200) planes of Au, respectively, suggesting the Au/CBO composite photocatalyst was successfully prepared [24]. The UV–vis absorption spectra were recorded to discuss the optical properties of the photocatalysts (Figure 2b). Evidently, the absorption edge of pure CBO extended to 700 nm, and according to the Kubelka–Munk function [29] and the plot of (α*h*ν)^2^ as a function of *h*ν (Figure S1, Supporting Information File 1), the band gap (*E*_g_) of CBO could be estimated to be 1.76 eV, which is consistent with the value reported in [30]. All of the Au/CBO composites exhibited a better absorption than pristine CBO in the ultraviolet and visible regions, revealing the influence of the SPR effect of Au NPs in the composite system.

**Figure 3:**
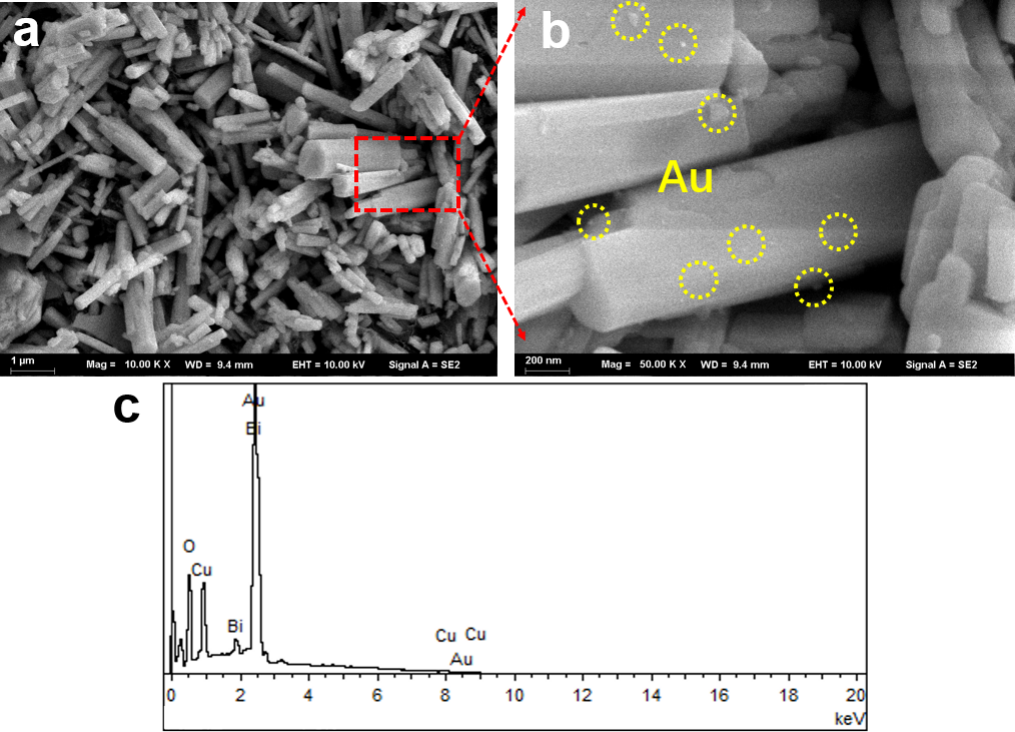
(a, b) SEM images and (c) EDX spectrum of the 2.5 wt % Au/CBO composite.

The morphological features of Au/CBO composite were characterized by using SEM. As can be seen in Figure S2 (Supporting Information File 1), pure CBO exhibits a rod-like structure with a length in the range of 2–6 μm and an average diameter of about 200 nm. After coating the Au NPs, the shape of CBO is not changed (Figure 3a). The enlarged SEM image in Figure 3b shows that the loaded Au NPs (the bright spots marked by the yellow circles) are uniformly distributed on the surface of the CBO microrods. EDX data in Figure 3c show the presence of Cu, Bi, O and small amounts of Au in the composite, further demonstrating that the 0D Au NPs have been loaded on the 1D CBO microrods. Additionally, the EDX–SEM mapping images (Figure S3, Supporting Information File 1) of Au/CBO also confirm this result.

**Figure 4:**
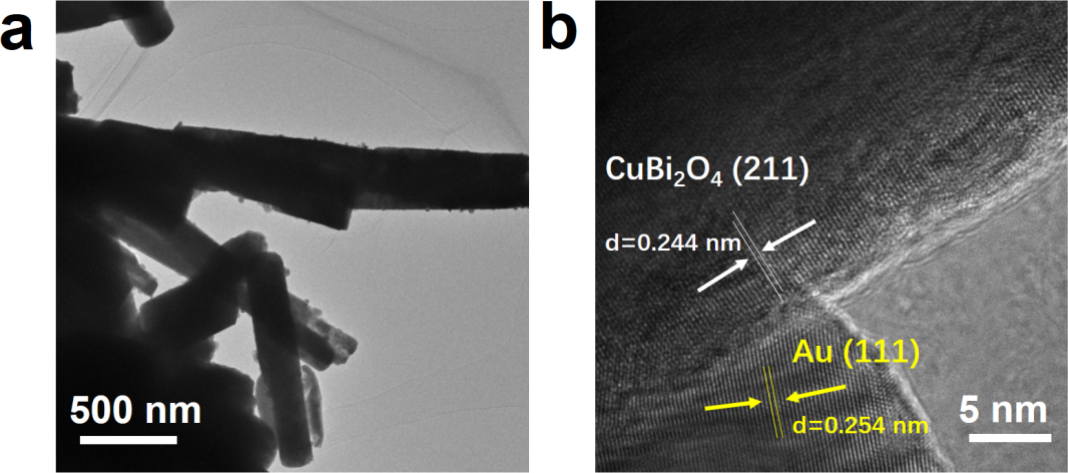
(a) TEM and (b) HRTEM images of the 2.5 wt % Au/CBO composite.

Also, TEM and HRTEM images were recorded. As shown in Figure 4a, Au NPs with a size of 20–30 nm were found to be uniformly dispersed on the surface of the CBO microrods. The HRTEM image of Au/CBO sample, presented in Figure 4b, provided detailed information about the 0D/1D heterostructure. The lattice spacings of 0.244 and 0.254 nm corresponded to the (211) and (111) crystallographic planes of CBO and Au, respectively [24,28]. Furthermore, a distinguishable interface and continuous lattice fringes can be observed between Au and CBO, revealing that a close heterojunction was formed in the composite photocatalyst.

**Figure 5:**
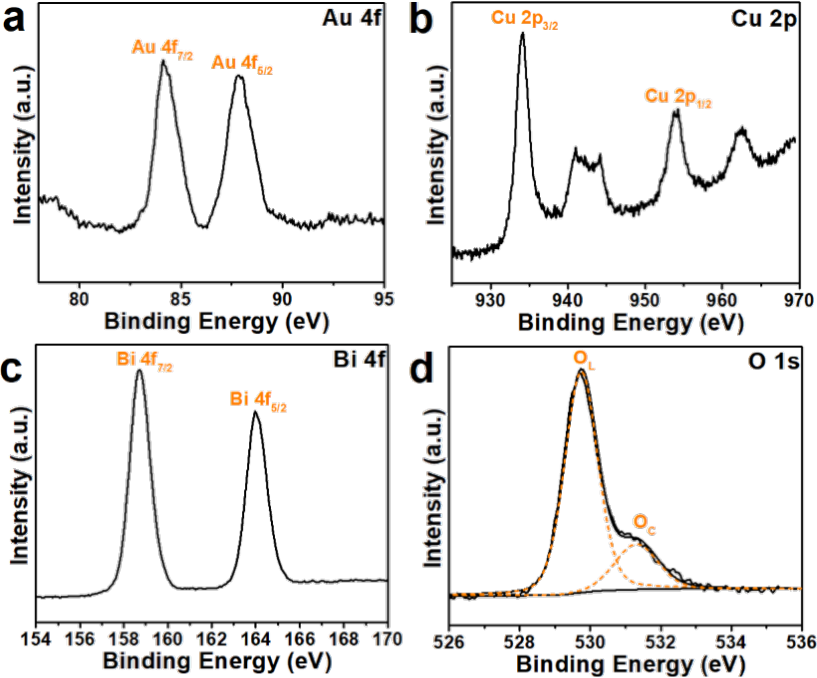
XPS high-resolution spectra of the 2.5 wt % Au/CBO composite: (a) Au 4f; (b) Cu 2p; (c) Bi 4f and (d) O 1s.

The chemical and electronic states of the elements on the Au/CBO surface were characterized by XPS. In the Au 4f XPS spectrum (Figure 5a) peaks at binding energies of 84.1 and 87.7 eV pertaining to Au 4f_7/2_ and Au 4f_5/2_, respectively, indicate that the state of Au on Au/CBO is Au^0^ [10]. The peaks at 934.1 and 953.8 eV in Figure 5b can be ascribed to Cu 2p_3/2_ and Cu 2p_1/2_, respectively [12]. The two peaks at 942.3 and 962.6 eV are satellite peaks of Cu 2p, further confirming that copper in the composite exist in a divalent state [31]. In the Bi 4f region (Figure 5c), two peaks at 158.5 and 164 eV are assigned to the Bi 4f_7/2_ and Bi 4f_5/2_, respectively, which affirms Bi^3+^ in CBO [32]. Figure 5d shows the XPS spectrum of O 1s composed of two main peaks at 529.8 and 531.6 eV [33–34]. The main peak at 529.8 eV be can attributed to lattice oxygen, and the higher-energy peak (531.6 eV) might be attributed to hydroxy groups on the surface [35–36].

**Figure 6:**
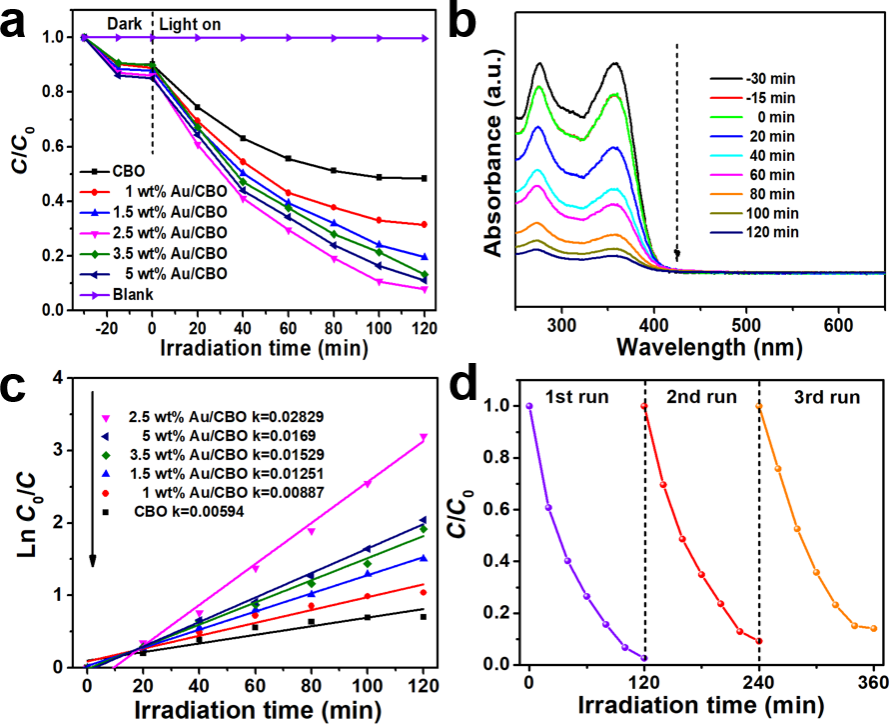
(a) TC degradation dynamics under visible-light irradiation. (b) Changes of the characteristic absorption of TC when using 2.5 wt % Au/CBO as a photocatalyst. (c) First-order kinetics of the TC degradation curves using different photocatalysts. (d) Three consecutive runs of TC degradation over 2.5 wt % Au/CBO photocatalyst.

The photocatalytic activity of the prepared photocatalysts was investigated by the degradation of TC under visible-light irradiation (λ > 420 nm). *C* and *C*_0_ are the final and the initial concentration of TC solution (measured by means of the absorbance at a wavelength of 357 nm). The blank experiment showed that the degradation rate of TC solution under visible-light irradiation is very low, indicating that the self-degradation of TC is negligible (Figure 6a). Pure CBO has a poor photocatalytic activity toward the degradation of TC, and only 52% TC was degraded after 120 min. The 0D/1D Au/CBO heterojunction photocatalysts showed a significant improvement in photocatalytic performance, demonstrating that Au NPs play an important role in the composites during the photocatalytic process. With increasing Au content, the photocatalytic degradation rate of TC firstly increased to a maximum of 93% (2.5 wt % Au/CBO), then decreased again when more Au NPs are added to the composite system. This phenomenon may be connected with excessive gold nanoparticles hindering the absorption of visible light. In addition, and the electron trapping also increases leading to a decreased photocatalytic activity [37–38]. Figure 6b shows the absorption of TC under the visible-light irradiation. When the solution is irradiated with visible light, the absorption of TC decreases faster. Figure 6c shows the apparent rate constants of all as-prepared materials with good linearity following first-order kinetics. The kinetic constant with added 2.5 wt % Au/CBO is 4.76 times higher than that with bare CBO. Recycling experiments were performed to test the stability of Au/CBO (Figure 6d). There was a slight decrease of photocatalytic activity after three cycles. This decrease of photocatalytic performance is mainly due to the mass of catalysts being inevitably lost in the recycling process [39].

**Figure 7:**
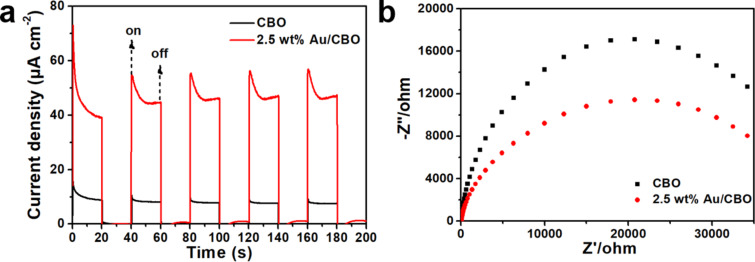
(a) Photocurrent response and (b) EIS spectra of bare CBO and 2.5 wt % Au/CBO composite under visible-light irradiation.

Photocurrent response and electrochemical impedance spectroscopy (EIS) measurements were carried out to obtain some insights into the photocatalytic activity of the Au/CBO composites. Figure 7a shows that both bare CBO and 2.5 wt % Au/CBO yield a photocurrent response under visible-light irradiation, but the photocurrent density of 2.5 wt % Au/CBO is much higher than that of the bare CBO. The combination of 0D Au NPs and 1D CBO microrods facilitates the generation of more effective photo-induced charges and accelerates the migration speed of electrons, resulting in a lower recombination rate of photogenerated carriers [40–43]. Figure 7b shows that a smaller high-frequency semicircle was obtained with 2.5 wt % Au/CBO than with pristine CBO, certifying the faster electron migration in 2.5 wt % Au/CBO.

**Figure 8:**
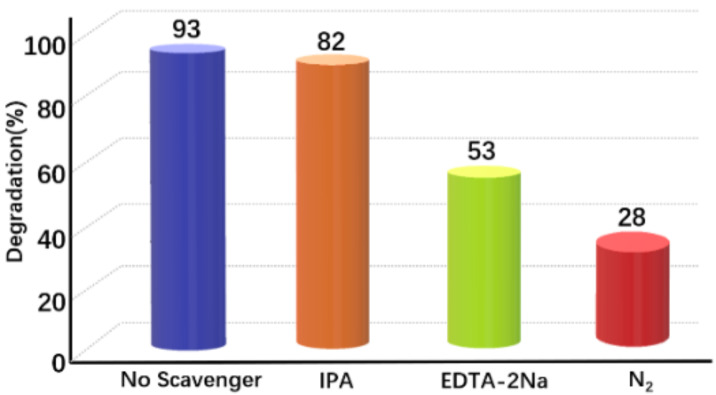
Trapping of reactive species during the photodegradation of TC over 2.5 wt % Au/CBO composite under visible-light irradiation.

In order to investigate the photocatalytic degradation mechanism of TC over the 0D/1D Au/CBO composite photocatalyst, the main radical species were detected through quenching experiments. Isopropanol (IPA), disodium ethylenediaminetetraacetic acid (EDTA) and nitrogen (N_2_) were separately introduced during the TC degradation as scavengers of·OH^•^, holes (h^+^), and·O_2_^−^, respectively. The degradation rate of TC was significantly suppressed when EDTA or N_2_ were added, while no obvious effect was observed when IPA was added (Figure 8). This suggests that h^+^ and·O_2_^−^ are crucial active species during the photocatalysis.

**Figure 9:**
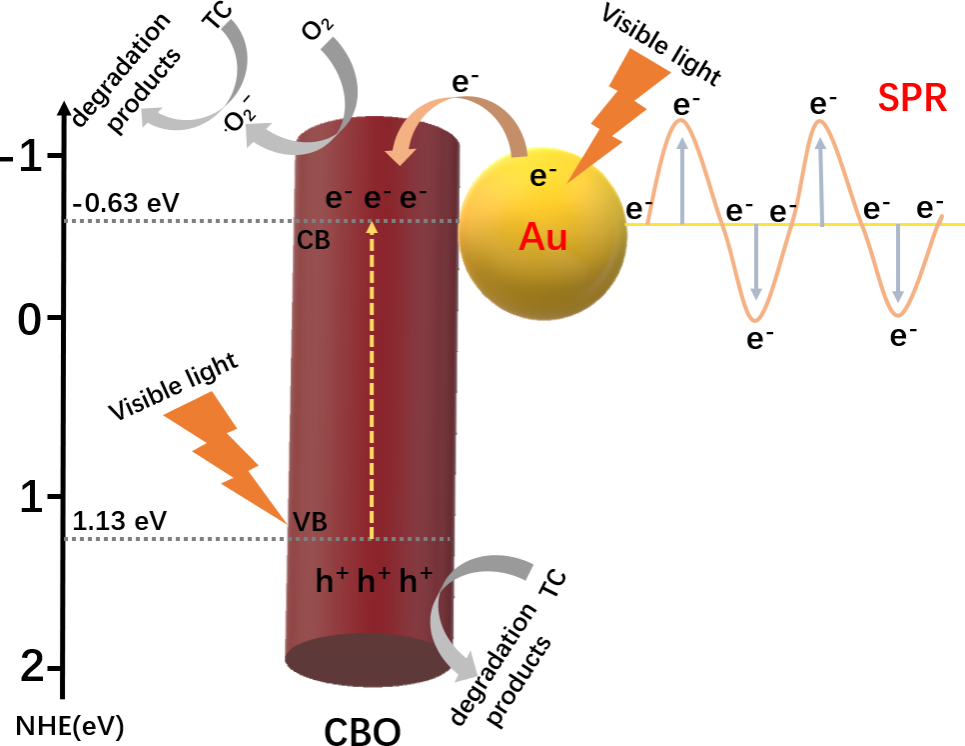
Possible photocatalytic reaction mechanism of the 0D/1D Au/CBO heterojunction photocatalyst during TC degradation under visible-light irradiation.

Based on the above experimental results, a possible photocatalytic mechanism was proposed (Figure 9). The valence band (VB) and conduction band (CB) positions of CBO can be estimated based on the following equation:


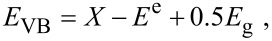


where *X* is the absolute electronegativity of the photocatalyst, *E*^e^ is the energy of the free electrons (4.5 eV) on the hydrogen scale, and *E*_g_ is the bandgap width of the photocatalyst. Thus, the VB and CB positions of CBO are calculated to be about 1.13 eV and −0.63 eV, respectively. Under irradiation with visible light, the electrons in the VB of CBO are excited and transferred to the CB. The Au NPs contacting the CBO microrods form a Mott–Schottky junction in the composite interface. In order to keep the Fermi level equal, the Fermi energy level of Au will shift closer to the CB of the CBO semiconductor, which can facilitate the separation of the photogenerated carriers [25]. Au NPs in the composite can be also excited under visible-light illumination due to the SPR effect. The hot electrons of Au NPs near the Fermi level can be excited to the SPR state and transferred to the CB of CBO to participate in the photocatalytic processes. Moreover, the higher specific surface area of 0D/1D Au/CBO composites can provide more active sites. Furthermore, based on the photocatalytic oxidation/reduction potential, the CB position of CBO is more negative than the reduction potential of O_2_/•O_2_^−^ (−0.046 eV vs NHE). Thus, O_2_ can be captured and reduced to •O_2_^−^ to degrade TC, while the holes in the VB of CBO can directly oxidize TC [10], which is consistent with the results of the trapping experiments (Figure 8). To sum up, the construction of 0D/1D heterojunction is conducive to the effective separation of photogenerated electron–hole pairs, thus greatly improving the photocatalytic activity of the semiconductor photocatalyst.

## Conclusion

0D/1D heterostructure Au/CBO composite photocatalysts were synthesized by a simple in situ thermal reduction–precipitation method. Due to the plasmon resonance effect of the Au NPs, the visible-light absorption of the CBO photocatalyst is enhanced. At the same time, the hot electrons excited by Au NPs can be injected into the conduction band of CBO, thus increasing the photogenerated charge in the reaction process. In addition, in the 0D/1D heterostructure the Au nanoparticles are dispersed more homogeneously on the surface of CBO microrods, which results in a higher specific surface area and an increased number of active sites. Furthermore, the recombination of photogenerated carriers is further suppressed and the photocatalytic activity is significantly improved. Our work suggests a rational structure design of efficient photocatalysts for environmental remediation.

## Supporting Information

File 1 Additional experimental data.
