# Supplementary material for: Construction of a 0D/1D composite based on Au nanoparticles/CuBi2O4 microrods for efficient visible-light-driven photocatalytic activity
Source: Beilstein J Nanotechnol. 2019 Jul 4;10:1360–7. doi: 10.3762/bjnano.10.134 (PMC6633693; doi:10.3762/bjnano.10.134)
Supplement: File 1 — Additional experimental data. [file Beilstein_J_Nanotechnol-10-1360-s001.pdf]

BEILSTEIN JOURNAL OF NANOTECHNOLOGY

Supporting Information

for

Construction of a 0D/1D composite based on Au nanoparticles/$CuBi_2O_4$ microrods for efficient visible-light-driven photocatalytic activity

Weilong Shi, Mingyang Li, Hongji Ren, Feng Guo, Xiliu Huang, Yu Shi and Yubin Tang

Beilstein J. Nanotechnol. **2019,** *10,* 1360–1367. doi:10.3762/bjnano.10.134

Additional experimental data

License and Terms: This is a supporting information file under the terms of the Creative Commons Attribution License (http://creativecommons.org/licenses/by/4.0). Please note that the reuse, redistribution and reproduction in particular requires that the authors and source are credited.
The license is subject to the *Beilstein Journal of Nanotechnology* terms and conditions: (https://www.beilstein-journals.org/bjnano)

Experimental

Characterization

X-ray diffraction (XRD) patterns were recorded using an X'Pert-ProMPD (Holand) D/max-γA X-ray diffractometer with Cu Kα radiation (λ = 0.154178 nm). Scanning electron microscopy (SEM) images were taken on a FEI-quanta 200 with an accelerating voltage of 20 kV. Transmission electron microscopy (TEM), high-resolution TEM (HRTEM) and high-angle annular dark-field scanning transmission electron microscopy (HAADF-SEM) were performed with a FEI-Tecnai F20 microscope operating at 200 kV. X-ray photoelectron spectroscopy (XPS) was carried out on a KRATOS Axis ultra-DLD X-ray photoelectron spectrometer with a monochromatized Al Kα X-ray source. UV–vis absorption spectra of the samples were recorded on a Lambda 750 (Perking Elmer) spectrophotometer in a range of 200 to 800 nm. The photocurrent–time (*i*–*t*) curves were recorded on a CHI 920C workstation (CH Instruments, Chenhua, Shanghai, China) using a standard three-electrode cell with a platinum wire as reference electrode and a saturated calomel electrode (SCE) as counter electrode. The *i*–*t* curves were measured at open-circuit potential in 0.5 M Na_2SO_4 electrolyte and a 300 W Xe lamp with UV-cutoff filter (λ > 420 nm) was used as the visible-light source. Electrochemical impedance spectroscopy (EIS) was carried out by using an alternating voltage of 5 mV amplitude in the frequency range from 10^5 Hz to 10^{-2} Hz.

Photocatalytic degradation of tetracycline

Briefly, 50 mg photocatalyst was suspended in 100 mL aqueous solution of 10 mg/L tetracycline (TC). The solution was stirred for 30 min in dark to ensure the establishment of adsorption equilibrium. Visible light (300 W, xenon lamp) was obtained by using cutoff filters to remove light of $\lambda < 420$ nm. At certain time intervals, 3 mL aliquots were extracted and centrifuged in the reaction process, then analyzed in the UV–vis spectrophotometer at a wavelength of 357 nm. For reproducibility and stability tests of the photocatalysts, the suspension was centrifuged after reaction and reused for repeated tests. The photocatalyst was collected and dried in a vacuum oven at 60 °C after each cycle of usage.

Capturing of active species

For the capturing tests, 2-propanol (IPA), disodium ethylenediamine tetraacetic acid (EDTA), and nitrogen (N_2) were used as scavengers to detect hydroxyl radicals (•OH), electron holes (h^+) and superoxide radicals ($\bullet O_2^-$), respectively. The above scavengers were added during the photocatalytic degradation of TC.

Additional Figures

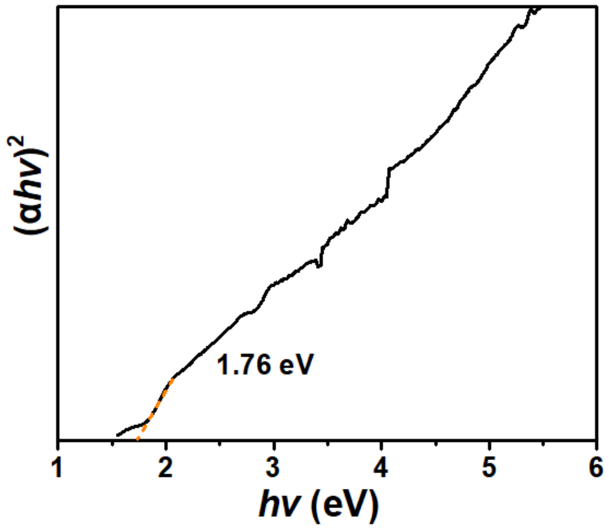

Figure S1: Plots of $(\alpha h\nu)^2$ as a function of $h\nu$ yielding the bandgap energy of pristine CBO.

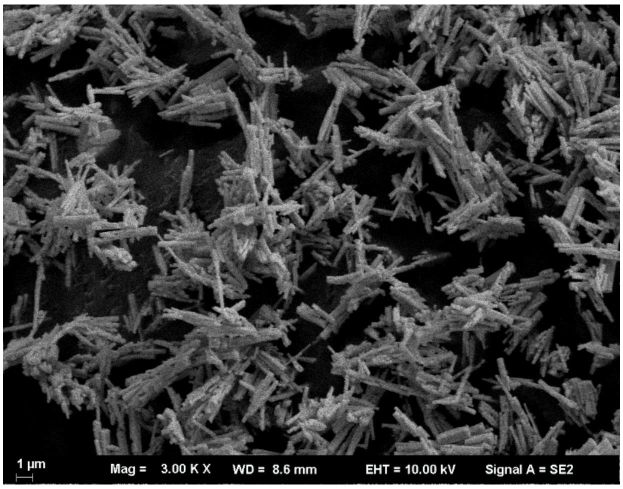

Figure S2: SEM image of pristine CBO.

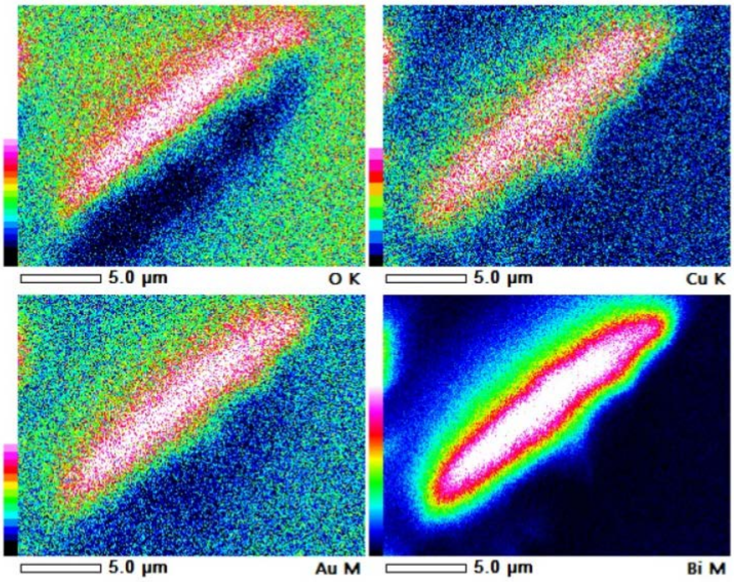

Figure S3: EDX–SEM maps of the 2.5 wt % Au/CBO composite.
